# COVID-19 Preventive Behaviors and Influencing Factors in the Thai Population: A Web-Based Survey

**DOI:** 10.3389/fpubh.2022.816464

**Published:** 2022-05-12

**Authors:** Kunwadee Rojpaisarnkit, Wonpen Kaewpan, Supa Pengpid, Karl Peltzer

**Affiliations:** ^1^Department of Public Health, Faculty of Sciences and Technology, Rajabhat Rajanagarindra University, Chachoengsao, Thailand; ^2^Department of Public Health Nursing, Faculty of Public Health, Mahidol University, Bangkok, Thailand; ^3^Department of Health Education and Behavioural Sciences, Faculty of Public Health, Mahidol University, Bangkok, Thailand; ^4^Department of Research Administration and Development, University of Limpopo, Polokwane, South Africa; ^5^Department of Psychology, College of Medical and Health Sciences, Asia University, Taichung, Taiwan

**Keywords:** COVID-19, preventive behaviors, face mask wearing, hand washing, physical distancing

## Abstract

**Objectives:**

To identify factors influencing COVID-19 preventive behaviors among the Thai population.

**Methods:**

A cross-sectional web-based survey was used. A total of 6,521 Thai people completed the survey. The multiple linear regression analysis was performed to identify factors that influenced coronavirus disease 2019 (COVID-19) preventive behaviors. The Predisposing, Reinforcing, and Enabling Constructs in Educational Diagnosis and Evaluation (PRECEDE) model was applied to propose factors influencing COVID-19 preventive behaviors.

**Results:**

The factors that mostly influenced COVID-19 prevention behaviors when controlling for the other variables are social support (β = 0.173, *p* < 0.001) follow by age (β = 0.162, *p* < 0.001), flu-like symptoms (β = 0.130, *p* < 0.001), gender (β = −0.084, *p* < 0.001), perceived risk of exposure (β = 0.035, *p* < 0.05), lock down policy (β = 0.029, *p* < 0.05), and residential area (β = −0.027, *p* < 0.05), respectively. These factors explained 52% of the COVID-19 preventive behaviors in Thai population.

**Conclusion:**

The result of this study was a foundation for further studies on different groups of people to develop different strategies to adopt preventive behaviors to reduce the spread of the COVID-19.

## Introduction

The coronavirus disease 2019 (COVID-19) is a respiratory disease that was first found in December 2019 in Wuhan, China, and quickly spread all over the world (1). The World Health Organization (WHO) declared COVID-19 a “Public Health Emergency of International Concern” on January 30, 2020, and later the WHO declared it a global pandemic on March 11, 2020 (2, 3). COVID-19 is continuing to spread throughout the world with more than 4.9 million deaths in October 2021 in almost 200 countries (4, 5). Thailand was the first country outside of China to detect a case of COVID-19 (3). In Thailand, from January 3, 2020 to September 2021, more than 1 million confirmed cases of COVID-19 and more than 113,000 deaths were, reported to WHO (4). As a result, Thai governments and related agencies have made efforts to control the spread of the disease.

The COVID-19 prevention efforts are heavily dependent on behavioral change and maintenance to control and reduce coronavirus transmission (6). Based on the concept of awareness of doing 3M (3 Movement) or three health protocols to avoid the spread of COVID-19, three important behaviors to reduce the spread of COVID-19 are hand washing, wearing a mask, and physical distancing (7, 8). These main behaviors make people the world over susceptible to COVID-19 and have become more critical than other behaviors (9).

Previous research found that consistent wearing of masks, hand washing, and physical distancing can protect against COVID-19 (8). Hand hygiene is one of the most important activities to stop the transmission of infection and prevent the spread of disease (10, 11). Moderate frequency hand washing (6–10 times per day) or regular hand washing with soap, hand sanitizer, gel, or spray alcohol can reduce the personal risk of developing coronavirus infection (12). Wearing face masks properly was strongly associated with a significant decrease in the risk of respiratory infections (8, 13–15). Last, physical distancing is one of the dominant habits in reducing disease transmission according to disease prevention guidelines (16).This study aimed to determine factors that influenced the COVID-19 preventive behaviors among Thai people and examine the association between the COVID-19 preventive behaviors and variables within the PRECEDE model (17). Rojpaisarnkit's study (18) utilized the PRECEDE model to propose factors that influenced disease prevention behaviors, and the study found that individual attribute factors, predisposing factors, enabling factors, and reinforcing factors that affect COVID-19 disease prevention behaviors (18). Therefore, in this study, the following factors were selected according to the PRECEDE model which included personal characteristics (e.g., gender, age, education, career, residential area and flu-like symptoms), predisposing factors (i.e., perceived risk of exposure to disease), enabling factors (i.e., lock down policy), and reinforcing factors (i.e., social support).

The results of the study will provide useful information on relevant factors associated with COVID-19 preventive behaviors that can be used in the development of an intervention to promote COVID-19 and other respiratory disease prevention behaviors in Thai people.

## Methods

Data used in this study were secondary data under the project title 'The assessment of psychosocial and behavioral response and compliance to restriction measures to prevent and control COVID-19: A series of rapid survey 'The questionnaire was developed based on a review of the literature on restriction measures to prevent and control COVID-19 and related theories. At first, the questionnaire was designed to be appropriate for use in online surveys and to remove bias from survey data which were reviewed for suitability by five experts. The conceptual framework of this research was developed based on a review of the theoretical concepts of the PRECEDE model and was used as a guide for selecting variables consistent with the research framework from the secondary data collected. Data integrity was verified, optimized, recoded for statistical analysis, and was examined before final analysis. Some questions were selected from the full survey of the above-mentioned project to examine the association between COVID-19 preventive behaviors (i.e., hand washing, face mask-wearing, and physical distancing) and other variables which included personal characteristics, perceived risk of exposure to disease, lock down policy, compliance with the measures of family members and the loss of a close person or social support which selected based on the PRECEDE model. The content validity of the questionnaire was checked by five experts, and the reliability with Cronbach's alpha scores was 0.702.

Data were collected using a web-based survey using a Google form questionnaire. The results of a cross-sectional study have been published elsewhere. Totally 6,521 Thai people completed an online survey from one survey between March and May 2020, inclusively (110 incomplete questionnaires were eliminated, representing 1.67%). For people who do not have the aptitude for answering google surveys form, they can let others help in answering. At the time of data was collected, it was found that in Thailand there were ~1–59 deaths from coronavirus per day, and about 40–4,000 people were infected per day (19).

Descriptive statistics were used to describe the participants' demographic characteristics by using frequency, percentage, arithmetic mean, and standard deviation. Bivariate correlations analysis was used to examine the relationship between each independent variable. To examine factors associated with COVID-19 preventive behavior, step-wise multiple linear regression analysis was performed. All data were analyzed, using Statistical Package for Social Sciences (SPSS) version 24. *P*-values of <0.05 were considered statistically significant.

## Results

Participants' demographic characteristics are presented in Table 1. Most of participants were female (64.4%), age between 30–49 years (48.8%), graduated bachelor degree (51.2%), worked as government services staff (36.8%), lived in urban area (52.4%), did not have flu like symptoms in the past week (84.1%), and did not have high risk of exposure to diseases in everyday life (69.3%) (Table 1).

**Table 1:** Participant's demographic characteristics.

**Variables**	**Number**	**Percentage (%)**
Gender		
Male	2,322	35.60
Female	4,199	64.40
Age (yrs.)		
15–19	316	4.80
20–29	1,225	18.80
30–39	1,589	24.40
40–49	1,589	24.40
50–59	1,429	21.90
60 and above	373	5.70
*Mean ± SD = 40.22 ± 12.80, Median 40.00, Min 15, Max 100*		
Level of education		
Primary school	203	3.10
Secondary school	1,388	21.30
Bachelor degree	3,339	51.20
Postgraduate	1,591	24.40
Career		
Not working, job less	950	14.60
Private business	759	11.60
General employee artisan	741	11.40
Government services	2,403	36.80
Farmers agricultures	111	1.70
Employee in organization	1,557	23.90
Residential area		
Rural	1,273	19.50
Suburban	1,829	28.10
Urban	3,419	52.40
Flu like symptoms in the past week		
Not have	5,483	84.1
Have mild symptoms	816	12.5
Have moderate symptoms	190	2.9
Very symptomatic	32	0.5
Perceived risk of exposure to diseases in everyday life		
Very little risk (score ≤ 12)	1,550	23.8
Little risk (score 13–19)	1,646	25.2
Moderate risk (score 20–23)	1,323	20.3
High risk (score ≥24)	2,002	30.7
*Mean ± SD = 18.24 ± 7.03, Median 20.00, Min 1, Max 32*		
Perception of lock down policy		
Yes	5,216	80.0
No	1,305	20.0
Social support		
Yes (score 6–10)	5,764	88.4
No (score 1–5)	757	11.6
*Mean ± SD = 8.07 ± 2.12, Median 8.00, Min 1, Max 10*		

The results of the bivariate correlation analysis between independent variables did not appear multicollinearity (coefficient value (*r*) ranging from −0.001 to 0.426) (20), as presented in Table 2. There were seven factors influencing COVID-19 preventive behaviors which included social support, age, flu-like symptoms, gender, perceived risk of exposure, lockdown policy, and residential area, respectively. It was found that social support had the greatest influence and was included in the step 1 of model for multiple regression analysis, followed by age, flu like symptoms, gender, perceived risk of exposure, lock down policy, and residential area. However, education and career were not significantly associated COVID-19 prevention behaviors and these two variables were not included in multiple regression analysis. Table 3 presented the multiple regression analysis of factors that influenced COVID-19 preventive behaviors. Regression coefficients (B) and Standardized coefficients (Beta) for COVID-19 prevention behavior predictors by stepwise approach are presented in Table 4. The result found that social support was the most influencing factor for COVID-19 prevention behaviors when controlling for the other variables was (β = 0.173, *p* < 0.001) follow by age (β = 0.162, *p* < 0.001), flu like symptoms (β = 0.130, *p* < 0.001), gender (β = −0.084, *p* < 0.001), perceived risk of exposure (β = 0.035, *p* < 0.05), lock down policy (β = 0.029, *p* < 0.05), and residential area (β = −0.027, *p* < 0.05), respectively.

**Table 2:** Bivariate correlation analysis of the relationship between independent variables.

	**Covid prevention behavior**	**Age**	**Gender**	**Education**	**Career**	**Residential area**	**Flu like symptoms**	**Perceived risk**	**Lock down policy**	**Social support**
Covid prevention behavior	1.00	0.199	−0.100	0.092	0.032^**^	−0.002^**^	0.180	0.026^**^	0.039^**^	0.228^**^
Age		1.00	0.018	0.283^**^	0.074^**^	0.123^**^	−0.033^**^	0.135^**^	0.011^**^	0.147
Gender			1.000	−0.173^**^	−0.010	−0.063^**^	0.024	−0.008	−0.043	−0.110
Education				1.000	0.207^**^	0.204^**^	0.060^**^	0.062^**^	0.046	0.057
Career					1.000	0.065^**^	0.426^**^	0.007	−0.019	0.019
Residential area						1.000	0.030^*^	−0.013	0.036^*^	−0.001^**^
Flu like symptoms							1.000	−0.009	0.024	0.157
Perceived risk								1.000	−0.059	0.013
Lock down policy									1.000	0.031^**^
Social support										1.00

** *Correlation is significant at the 0.01 level (2-tailed)*.

* *Correlation is significant at the 0.05 level (2-tailed)*.

**Table 3:** Multiple regression analysis of factors that influenced COVID-19 preventive behaviors.

**Factor**	**R**	**R Square**	**Adjusted R Square**	**Std. Error of the Estimate**	**Change statistics**				
					**R Square Change**	**F Change**	**df 1**	**df 2**	**Sig. F Change**
Social support	0.228^a^	0.052	0.052	1.269	0.052	357.227	1	6,519	<0.001
Age	0.283^b^	0.080	0.080	1.250	0.028	198.292	1	6,518	<0.001
Flu like symptoms	0.310^c^	0.096	0.096	1.239	0.016	118.258	1	6,517	<0.001
Gender	0.321^d^	0.103	0.103	1.235	0.007	48.760	1	6,516	<0.001
Perceived risk	0.323^e^	0.104	0.103	1.234	0.001	7.590	1	6,515	0.006
lock down policy	0.324^f^	0.105	0.104	1.234	0.001	5.503	1	6,514	0.019
Residential area	0.325^g^	0.106	0.105	1.233	0.001	5.206	1	6,513	0.023

a *Predictors: (constant), social support*.

b *Predictors: (constant), social support, age*.

c *Predictors: (constant), social support, age, flu like symptoms*.

d *Predictors: (constant), social support, age, flu like symptoms, gender*.

e *Predictors: (constant), social support, age, flu like symptoms, gender, perceived risk of exposure*.

f *Predictors: (constant), social support, age, flu like symptoms, gender, perceived risk of exposure, lock down policy*.

g *Predictors: (constant), social support, age, flu like symptoms, gender, perceived risk of exposure, lock down policy, residential area*.

**Table 4:** Regression coefficients (B) and Standardized coefficients (Beta) for COVID-19 prevention behaviors.

**Factor**	**Unstandardized Coefficients**		**Standardized Coefficients**	***t***	**Sig**.	**95% Confidence Interval for B**	
	**B**	**Std. Error**	**Beta**			**Lower Bound**	**Upper Bound**
(Constant)	3.968	0.144		27.463	<0.001	3.685	4.251
(Constant)	0.106	0.007	0.173	14.363	<0.001	0.092	0.121
Social support	0.016	0.001	0.162	13.432	<0.001	0.014	0.019
Age	0.341	0.031	0.130	10.874	<0.001	0.280	0.403
Flu like symptoms	−0.229	0.032	−0.084	−7.106	<0.001	−0.292	−0.166
Gender	0.006	0.002	0.035	2.973	0.003	0.002	0.011
Perceived risk	0.093	0.038	0.029	2.425	0.015	0.018	0.168
Lock down policy	−0.045	0.020	−0.027	−2.282	0.023	−0.084	−0.006

From the multiple regression analysis, social support, age, flu like symptoms, gender, perceived risk of exposure, lock down policy, and residential area explained 10.6% of the COVID-19 preventive behaviors in Thai population. The multiple regression equation was Y= 3.968 + 0.173X9 + 0.162X1 + 0.130X6 − 0.084X2 + 0.035X7 + 0.029X8 − 0.027X5.

## Discussion

From the overview of the model, it was found that the R-Squared and Adj R-square value which is a descriptive variation was low. In general, though a high R-Squared value means that the statistical model is good or appropriate for the data. However, just because a low R-Squared value may not mean that the statistical model is not always good (21). Especially in predicting human behavior since humans are more difficult to predict compared to physical processes (22). In the case of COVID-19 prevention behaviors in this study, the low R-Squared values may be because at the time of data collection, Thai people were confused with the information about disease and the situation about COVID-19 in Thailand that affected COVID-19 prevention behaviors. However, due to the low R-Squared value, if further research is to be conducted in the future, it is necessary to revisit the variable selection guidelines. This is because there may be other variables that are important to COVID-19 prevention behaviors of Thai people's that should be further studied.

From the results found that different individual characteristics, predisposing factors, enabling factors and reinforcing factors had a significant association with COVID-19 preventive behaviors. The discussion of each variable was as following:

### Social Support

Social support had the greatest influenced on COVID-19 preventive behaviors, consistent with previous study that personal hygiene habits of close person or compliance with the measures of family members and close person was associated with COVID-19 preventive behaviors (23). Communication strategies may need to emphasize the role that friends and families can play in helping to promote COVID-19 preventive behaviors. Further studies should examine which kind of message can most effectively encourage people to adopt preventive behaviors with COVID-19.

### Age

Age influenced COVID-19 preventive behaviors, consistent with previous studies that higher age was a predictor for higher adherence of COVID-19 preventive behaviors (24). Another study also found that older people were also likely to embrace protective behaviors (25). In addition, previous studies found significant association between ages and face mask-wearing (26–29). Moreover, worsening health behavior change during COVID-19 lock down was associated with being younger (30). Therefore, further study may explore how younger and older people are difference when they adopt COVID-19 preventive behaviors. Different strategies may need to motivate younger and older people to adopt preventive behaviors to reduce spread of COVID-19.

### Flu-Like Symptoms

Experiencing flu-like symptoms influenced COVID-19 preventive behaviors, consistent with previous study that experiencing flu-like symptoms was associated with poor adherence of COVID-19 preventive behaviors (24). Individuals who experience flu-like symptoms may be more at risk of becoming infected; therefore, continued compliance with preventive behaviors is recommended to reduce the transmission of COVID-19.

### Gender

Gender influenced COVID-19 preventive behaviors, consistent with previous studies that there were significant association between face mask wearing and gender. Previous studies found that females wore masks correctly than male (31–33). Another study also found that gender is an important predictor of hand hygiene practices (34). Moreover, worsening health behavior change during COVID-19 lock down was associated with being female (30). Female may have a greater perception of the severity of the COVID-19 pandemic and greater adherence to prevention measures (35). Thus, further study may explore gender difference in adoption of COVID-19 preventive behaviors which may inform policy intervention.

### Perceived Risk of Exposure to Disease

The perceived risk of exposure to the disease influenced the preventive behaviors of COVID-19, consistent with a previous study that risk perception was associated with an increase in overall protective behaviors of COVID-19 (36). In addition, perceived risk of exposure to disease was significantly associated with adoption of preventative health behaviors in all 10 countries across Europe, America, and Asia (2). Moreover, there was a positive relationship between severity perception and preventive behaviors (37). Higher risk perception was subsequently associated with higher frequency/probability of practicing preventive behaviors (38), and people's risk perception has shaped their protective behaviors during the lockdown (39). However, some studies found that perceived severity was not significantly predictive of COVID-19 preventive behaviors (40). Understanding the risk perception of the COVID-19 pandemic among Thais could help policymakers, and health professional to develop better communication strategies that promote the adoption of COVID-19 preventive behaviors.

### Lock Down Policy

The lockdown policy influenced the preventive behaviors of COVID-19, consistent with the previous study that the impact of the COVID-19 lockdown was significantly associated with health-related behaviors (41). Another study also found that people has shaped their protective behavior during the lockdown (39). People have responded differently to lockdown policy, and these differences might be associated with a number of factors such as personal, social, cultural, mental, and economic variables (42). Therefore, understanding individual differences toward lockdown policy among Thais could help policy makers and healthcare professionals develop effective strategies to promote the adoption of COVID-19 preventive behaviors.

### Residential Area

Residential area influenced COVID-19 preventive behaviors, consistent with the previous study that regional differences or living location was associated with the adoption of protective behaviors (25). Rural residents were significantly less likely to worn a mask in public area compared to urban residents (43). Another study also found respondents in urban areas had more accurate knowledge of disease patterns and had adopted more protective behaviors than rural respondents (44). Several factors may contribute to residential area difference such as health access, restriction measures. Thus, difference strategies are needed to improve COVID-19 preventive behaviors in urban and rural area. For example, effective communication strategies that meet rural populations' unique needs can be an effective strategy to adopt preventive behaviors to against COVID-19.

### Education

Education was not significantly associated with COVID-19 preventive behaviors, consistent with previous study that higher educational level did not affect the quality of personal hygiene and healthy practice behaviors during the COVID-19 outbreak (45). In contrast, other studies found that education was significantly associated with face mask wearing (29, 46) and educational differences were found in the adoption of protective behaviors (25). Health literacy and access to COVID-19 information were associated with engaging in more COVID-19 preventive health actions (47). Therefore, to improve the adoption of COVID-19 preventive behaviors, policy maker and health professionals should plan to promote health literacy focusing on prevention and access to COVID-19 relevant information sources.

### Career

Career was not significantly associated with COVID-19 preventive behaviors, consistent with a previous study that the wearing of masks in COVID-19 situations was not associated with occupation (48). In contrast, another study found that career was significantly associated with face mask wearing (49). Further study should examine how career play a role in COVID-19 preventive behaviors and these differences might be associated with several factors such as knowledge and economic variables.

The results of this study provided information on factors that influenced the prevention behaviors of COVID-19 disease among Thai people, which was a basis for further studies in different groups of people in order to develop different strategies to adopt preventive behaviors to reduce the spread of COVID-19.

## Data Availability Statement

The original contributions presented in the study are included in the article/supplementary materials, further inquiries can be directed to the corresponding author.

## Ethics Statement

The studies involving human participants were reviewed and approved by Faculty of Social Sciences and Humanities, Mahidol University. The patients/participants provided their written informed consent to participate in this study.

## Author Contributions

KR, WK, SP, and KP contributed to the design and implementation of the research. KR analyzed the results. WK and KR wrote the manuscript with input from all authors. All authors contributed to the article and approved the submitted version.

## Funding

Scholarly publishing was supported by Faculty of Public Health, Mahidol University.

## Conflict of Interest

The authors declare that the research was conducted in the absence of any commercial or financial relationships that could be construed as a potential conflict of interest. The reviewer PN declared a shared affiliation with the author WK to the handling editor at the time of review.

## Publisher's Note

All claims expressed in this article are solely those of the authors and do not necessarily represent those of their affiliated organizations, or those of the publisher, the editors and the reviewers. Any product that may be evaluated in this article, or claim that may be made by its manufacturer, is not guaranteed or endorsed by the publisher.
